# Clinical Utility of Epigenetic Changes in Pancreatic Adenocarcinoma

**DOI:** 10.3390/epigenomes5040020

**Published:** 2021-09-27

**Authors:** Joyce K. Thompson, Filip Bednar

**Affiliations:** Department of Surgery, University of Michigan, Ann Arbor, MI 48109, USA; joycekt@med.umich.edu

**Keywords:** pancreatic adenocarcinoma, pancreatic cancer, DNA methylation, histone, epigenetics, diagnosis, screening, therapy

## Abstract

Pancreatic cancer is a molecularly heterogeneous disease. Epigenetic changes and epigenetic regulatory mechanisms underlie at least some of this heterogeneity and contribute to the evolution of aggressive tumor biology in patients and the tumor’s intrinsic resistance to therapy. Here we review our current understanding of epigenetic dysregulation in pancreatic cancer and how it is contributing to our efforts in early diagnosis, predictive and prognostic biomarker development and new therapeutic approaches in this deadly cancer.

## 1. Introduction

Pancreatic adenocarcinoma (PDA or pancreatic cancer here) is one of the deadliest malignancies with the 5-year survival rate in the United States just reaching 10% [1]. This remains so low because 80–85% of patients present with anatomically unresectable or metastatic disease at the time of their diagnosis with an average survival of 9–12 months [2,3]. Even patients that undergo a curative surgical resection will recur in the vast majority of cases. Systemic chemotherapy remains only marginally effective, although the response rates are very slowly improving with newer combination treatments [2,3]. In addition, the prevalence—how many people are diagnosed with PDA—continues to increase and will make PDA the second deadliest cancer by 2030 [4]. Alterations in DNA methylation, mutations in chromatin regulatory protein complexes and other dysregulated epigenetic mechanisms contribute to PDA aggressiveness. Here we review how our understanding of epigenetic dysregulation is beginning to contribute to new approaches for early diagnosis and treatment of pancreatic cancer.

## 2. Molecular Subtypes of Pancreatic Cancer

Extensive efforts over the past decade have begun to unravel the biological heterogeneity of PDA. Collison and colleagues first performed a transcriptional analysis of tumor epithelia from macro- and microdissected PDA and tumor cell lines [5]. This allowed the authors to group PDA into three main types—classical, quasi-mesenchymal and exocrine. Classical PDA type was KRAS-dependent and expressed epithelial gene signatures whereas the quasi-mesenchymal type upregulated mesenchymal genes. Exocrine-like PDA, as implied by its name, was the closest to enzyme-secreting acinar cells in terms of its transcriptome. Subsequent work has suggested that this subtype was more likely confounded by normal parenchymal contamination [6]. A computational deconvolution of bulk transcriptome data by Moffitt and colleagues also identified two primary epithelial subtypes in PDA—classical and basal-like [7]. Subsequent large scale next generation sequencing efforts through the International Cancer Genome Consortium (ICGC) and The Cancer Genome Atlas (TCGA) have expanded on these early efforts and solidified the concept of two primary epithelial PDA types—classical/pancreatic progenitor and basal-like/squamous [6,8]. More recently, Lomberk and colleagues identified specific epigenetic states and associated transcriptional regulatory networks that stabilize the two primary transcriptome-defined PDA subtypes [9]. Together, these findings provide a molecular framework to consider tumor evolution and heterogeneity within our patients with direct implications for diagnosis and therapy.

Transcriptome classification of PDA has also been complemented by whole exome or whole genome sequencing to define oncogenic drivers and genomic structural alterations including insertions/deletions, copy number alterations and whole genome duplications [6,8,10]. Along with mutations in the classical PDA-associated oncogenes and tumor suppressors like KRAS, TP53, CDKN2A and SMAD4, the sequencing efforts also uncovered mutations in many chromatin modifying enzymes and complexes. These include mutations in SWI/SNF chromatin remodeler complex subunits such as ARID1A/B, SMARCA4/BRG1, PBRM1/BAF180, histone lysine demethylase KDM6A and histone lysine methyltransferases MLL2/3/4 [6,8,10]. Functional mechanistic studies of these chromatin regulators in genetically engineered mouse models of PDA have now implicated many of them in the neoplastic transformation of the pancreas [11,12,13,14,15,16,17,18].

More recent work has now begun to integrate how mutations in these chromatin regulators correlate with tumor evolution, genomic structural evolution and the transcriptional subtypes of PDA in human patients [19,20]. Iacobuzio-Donahue and colleagues undertook a detailed genomic, transcriptomic and histologic analysis of human PDA in their rapid autopsy cohorts [20]. This detailed analysis allowed for the inference of tumor evolutionary phylogenetic relationships within distinct regions of the primary tumors and metastases from the same patient. The authors observed a clear correlation between the histological squamous PDA regions with the basal/squamous transcriptome subtype. In comparison, the glandular histology was marked by the classical subtype transcriptome. Through phylogenetic analysis the squamous/basal areas were inferred to arise in what were initially classical glandular subtype tumors. This evolution from classical to basal-like areas also correlated with increased genomic structural rearrangements and mutations in multiple chromatin epigenetic regulators. A concurrent work by Notta and colleagues analyzing the COMPASS trial participants independently reached similar conclusions by analyzing the transcriptome changes along with structural genomic alterations between temporally distinct primary tumors and recurrences or metastases [19]. In addition, single cell transcriptomic analysis demonstrates the coexistence of classical and basal-like transcriptome-containing cells within a single tumor [19]. A key paradigm that has emerged from this work is the idea that individual PDA tumors really exist on a continuum of transcriptional phenotypes and that progression from the classical to basal-like type correlates with specific structural genome changes, chromatin dysregulation, alteration of the functional transcriptome and increased aggressiveness with propensity to metastasize [19,20]. Complementing this evolutionary perspective is the observation that driver mutations in primary tumors do not differ markedly between primary tumors and metastases within the same patient [21,22]. In addition, in each patient, primary PDA tumors and metastases differed mainly in their epigenetic adaptations to the new metastatic niche and its related metabolic demands [23]. This data implicates chromatin and epigenetic regulation in PDA progression and metastatic spread and validates ongoing efforts to develop diagnostic and therapeutic clinical tools for PDA based on the epigenetic or chromatin state of the tumor.

## 3. Epigenetic Modifications in Early Diagnosis and Screening of Pancreatic Neoplasia

### 3.1. Current Screening and Diagnostic Approaches in Pancreatic Cancer

A conceptual framework, credited to Dr. George Crile, a cancer surgeon in the mid-20th century, proposed that preneoplastic and neoplastic lesions can be classified as ‘turtles’, ‘rabbits’ or ‘birds’ based on their long-term biologic behavior after diagnosis [24,25]. The ‘turtles’ represent slow-growing indolent lesions that will never progress to a highly metastatic and aggressive state. The ‘rabbits’, in contrast, are localized at the time of diagnosis, but carry the potential to become lethal metastatic cancers if left untreated. The ‘birds’ are the aggressive tumors that have metastasized and are highly unlikely to be cured even with aggressive surgery and systemic therapy. In the case of pancreatic cancer, vast majority of the diagnosed tumors currently behave like ‘birds’—they recur and kill the patient, even if they are found early enough to be resected. It is the goal of the current screening and early diagnosis efforts for PDA to identify patients with high risk preneoplastic and early stage neoplastic lesions before they turn from a ‘rabbit’ to a ‘bird’ [26].

Diagnostic approaches to pancreatic cancer have to contend with its low prevalence within the general population. In 2017, approximately 79,000 patients with existing or newly diagnosed pancreatic cancer were living in the United States [27]. The approximate prevalence for the general US population was ~20/100,000 people. Given this low prevalence, a diagnostic test for pancreatic cancer will have to be essentially 100% sensitive—have the ability to identify every patient in a mixed population of healthy and sick patients —and 100% specific—define every healthy person as truly healthy. In a general population with a low prevalence of PDA, poorer performance would result in a high rate of false positive diagnoses leading to unnecessary further testing, exposure to possible complications and the stress associated with possibly having a lethal cancer.

One approach to overcome these stringent diagnostic test performance requirements is to identify patient populations with an increased prevalence or risk for cancer development. Pancreatic cancer arises from a multitude of preneoplastic lesions including pancreatic intraepithelial neoplasia (PanINs), mucinous cystic neoplasms (MCNs) and intraductal papillary mucinous neoplasms (IPMNs) [28]. In addition, genetic syndromes and familial variants that predispose patients to develop pancreatic cancer have been defined [29]. Current guidelines from the International Cancer of the Pancreas Screening Consortium (CAPS) recommend a multimodal approach with blood testing, MRI and endoscopic ultrasound to identify preneoplastic and early neoplastic lesions in these high risk, higher prevalence patients [30]. Variants of such screening protocols have been established over the past decade with some encouraging results [31]. Although these programs increase the rate of true positive diagnoses, the performance of the existing diagnostic tests still identifies many ambiguous lesions with uncertain malignant potential. In addition, current screening programs are not sensitive and specific enough to be deployed across the general population in a manner similar to colonoscopies for colon polyps and cancers. A complementary approach then is to develop new minimally invasive diagnostic tests with improved performance compared to the currently used protein biomarker and imaging tests. In the case of PDA, one promising path has been to leverage the dysregulated methylation of the tumor genome through a serum or plasma-based test.

### 3.2. Aberrant DNA Methylation as a Diagnostic Biomarker for Pancreatic Cancer

The observation that tumors display tissue-of-origin and tumor-specific CpG island methylation has been known for a long time [32,33]. In the context of pancreatic cancer, xenografts and tumor-derived cell lines initially demonstrated aberrant methylation of at least one tumor-related gene promoter from a 13-gene panel in ~60% of the tested samples [34]. The authors used sensitive methylation-specific PCR (MSP) to detect the altered CpG island methylation and suggested that this may translate well into a future early diagnosis test. Subsequent work using combinations of MSP with microarrays and laser capture microdissection has defined specific hypermethylation and hypomethylation changes not only in pancreatic cancers, but also in PanINs and IPMNs [35,36,37,38,39]. An interesting and consistent observation across multiple studies has also been that the magnitude of changes in methylation correlates with the level of dysplasia in the lesions. In addition, comparison of familial pancreatic cancer patient tumors with those from the general population revealed similar methylation patterns suggesting that any DNA methylation-based diagnostic approaches should be equally applicable across both of these PDA patient cohorts [40].

The initial assessment of DNA methylation changes in PDA and its precursors was done using small panels of genes and promoters preselected based on prior biological understanding of pancreatic cancer. However, these may not represent the best analytes to include in a future diagnostic test. To define an unbiased comprehensive list of targets with aberrant methylation in PDA, genome-wide approaches using methylation microarrays and RNA expression profiling were used [41,42,43]. Additional specificity in these studies was provided by treating pancreatic cancer cell lines with 5-aza-2′-deoxycytidine (decitabine)—a DNA methyltransferase inhibitor—and trichostatin A (TSA)—a histone deacetylase inhibitor to identify targets, whose expression changes with their methylation status [41]. These studies confirmed that both hyper and hypomethylation play a role on a genome-wide level in PDA with hypermethylation being the more prevalent alteration [43]. The functional studies with decitabine and TSA also identified a subset of genes, whose expression was altered with the change in their methylation status. In addition to protein transcripts, dysregulated expression due to hypomethylation was also seen for the miR-200 family of miRNAs, which are often overexpressed in pancreatic cancer and help maintain the epithelial phenotype by regulating the epithelial–mesenchymal transition in tumor cells [44,45,46]. Changes in methylation status on a genome-wide level contribute to pancreatic cancer pathology and their genome-wide assessment has allowed for the development of the next generation of diagnostic assays.

Along with more comprehensive identification of assay targets, the sample collection methods and inclusion of appropriate control populations has also evolved. The initial proof of dysregulated methylation was performed using directly biopsied or resected tumor specimens and cell lines [34,35,37,38,39]. Early clinical development and validation was done through collection of hormone-stimulated secretion of pancreatic juice [36,41,47]. Unfortunately, endoscopic acquisition of pancreatic juice requires high level of technical expertise, advanced endoscopic equipment and its sensitivity may be altered by methylation changes from the adjacent duodenal epithelium, which is often incidentally sampled during the procedure [36]. These considerations led to the development of plasma and serum-based DNA methylation assays since blood is easily obtainable in a reasonable quantity and is routinely collected during the diagnostic workup and treatment of pancreatic disease. Li and colleagues performed a tissue-based analysis of DNA methylation for the miR-200a/b miRNA and SIP1 loci in pancreatic tumors and cell lines [44]. They correlated this with miRNA expression levels in the serum of pancreatic cancer patients, pancreatitis patients and healthy controls. They identified hypomethylation of the tested loci in the tissue and demonstrated high levels of the miRNAs in the serum of both pancreatitis and PDA cohorts [44]. Subsequent efforts have continued to develop more sensitive and specific serum-based approaches to identify tumor-specific methylation changes for pancreatic cancer diagnosis.

### 3.3. Current Serum Based DNA Methylation Diagnostic Assays for Pancreatic Cancer

The transition to serum-based diagnostic tests along with technological advances in DNA isolation and next generation sequencing have led to new highly sensitive multi-analyte assays. These assays mirror the general trend of developing liquid biopsy approaches to define tumor heterogeneity, metastatic status and treatment response through assessment of multiple analytes in blood in other solid organ tumors [48]. Yi and colleagues used new methylation-on-bead technology with quantitative MSP to specifically screen serum circulating free DNA (cfDNA) for promoter methylation changes in the BNC1 and ADAMTS1 loci—two genes that were verified to be hypermethylated in pancreatic cancer [49]. The tested cohort included 42 pancreatic cancer patients and 26 healthy controls. The combined methylation panel had a sensitivity of 81% and specificity of 85%. These performance results were not adequate for full clinical use, but the study served as a proof of principle for the use of tumor-specific methylation in cfDNA in pancreatic cancer diagnosis. Testing of this assay in a separate prospectively enrolled cohort of early stage (I-IIB) resectable pancreatic cancer patients along with pancreatitis and healthy controls demonstrated elevated levels of methylation in both the pancreatic cancer and pancreatitis patients [50]. These results are important as they highlight the need for inclusion of pancreatitis controls in any diagnostic assay trials in the pancreatic cancer space since pancreatitis patients have often demonstrated similar results to pancreatic cancer patients in diagnostic testing. At this point, no liquid biopsy approach performs well enough to distinguish pancreatic cancer and pancreatitis patients with high sensitivity and specificity and continued inclusion of both of these groups of patients in future assay development is key.

A separate proof-of-principle study involving cfDNA methylation as a marker of tissue specific cell death was used in a cohort of early stage pancreatic cancer patients and other cancer patients and diabetes patients [51]. The assay targeted hypomethylation of the REG1A and CUX2 loci as these were specifically hypomethylated in normal acinar and ductal cells of the pancreas. The authors used methylation levels of the two loci alone and in combination with mutation status of KRAS—the primary oncogenic driver in pancreatic cancer—to study 42 pancreatic cancer patients. In this cohort, the methylation status had significantly higher sensitivity compared to the KRAS mutation status in identifying the cancer patients. Of note, 70% of the tested pancreatitis controls also demonstrated changes in cfDNA methylation consistent with the idea proposed by the authors that cfDNA methylation represents elevated tissue death or cell turnover in an organ under stress [51]. To improve the sensitivity of the cfDNA methylation approach, a new methylated cfDNA immunoprecipitation method combined with next-generation sequencing has now been tested [52]. The resulting approach had much higher sensitivity compared to cfDNA mutation profiling and was able to identify tumor-specific cfDNA methylation patterns that distinguished different tumor types. This characteristic would be particularly useful in clinical cases, where the primary site of disease is not known. The authors validated the assay in a separate cohort of 199 samples, which included 47 pancreatic cancer patients. The area under the receiver operating characteristic curve (AUROC) for the assay was 0.914–0.920 for the PDA cohort. The assay performed similarly well with early and late-stage PDA patients highlighting its potential clinical utility for early diagnosis in pancreatic cancer.

The key question that remains to be addressed is how to integrate analysis of epigenetic alterations within the tumor into the larger landscape of diagnostic approaches to pancreatic cancer. A parallel approach testing a combination of cfDNA and protein biomarkers in pancreatic patients demonstrated an impressive specificity of 99.5% [53]. The panel tested for the presence cfDNA KRAS mutations and serum levels of CA19-9, CEA, HGF and OPN—protein markers that are used clinically to follow pancreatic cancer patient treatment course or have been previously found to be dysregulated in pancreatic cancer patients. Although the sensitivity of this multi-analyte panel was still low, this work tested the conceptual idea of combining different classes of analytes—in this case cfDNA and circulating serum proteins—in a single assay. The approach was subsequently expanded to the Johns Hopkins CancerSEEK panel, which includes eight protein analytes and 61 PCR amplicons [54]. This panel was tested prospectively in 1005 cancer patients and 812 controls with sensitivity of ~70% and specificity >99% in the pancreatic cancer patients. In addition, since the test was also used to analyze patients with different primary tumors, the authors were able to define how well it could identify the primary site of disease. This is important since early-stage pancreatic cancers and preneoplasic lesions may not be amenable to imaging localization using current clinically available techniques. Combining cfDNA methylation testing with mutational and protein biomarkers, such as the CancerSEEK panel, has the potential to further improve test sensitivity and specificity and presents an exciting avenue to move forward in the field of pancreatic cancer early diagnosis.

It is notable that the development of DNA methylation-based diagnostics for PDA proceeded in parallel to our increasing understanding of the role of other epigenetic processes in PDA biology. At this point, there is not a significant overlap between our insight into PDA functional subtypes, how they are regulated and how they evolve and the diagnostic approaches to this deadly disease. Future efforts will need to better integrate these two areas of expertise to develop a new generation of early diagnostic tests.

## 4. Epigenetic Modifications as Biomarkers

### 4.1. PDA Subtypes as Prognostic and Predictive Entities

Useful clinical biomarkers fall into two categories—predictive and prognostic. Predictive biomarkers are measurable characteristics within a patient that correlate with the possibility of patient response to a particular therapy. Conversely, prognostic biomarkers define categories marked by different survival within the patient population. The previously defined PDA molecular subtypes—classical and basal-like/squamous—serve both of these functions. Multiple studies have demonstrated that patients with the basal/squamous subtype have poorer overall survival than those with the classical subtype [5,7,8,55]. Collisson and colleagues also performed the first predictive biomarker analysis related to these subtypes and found that the classical tumors are potentially more sensitive to erlotinib—an epidermal growth factor receptor (EGFR) kinase inhibitor, while the quasi-mesenchymal/basal-like tumors are sensitive to gemcitabine [5]. Molecular subtypes of PDA have also been found to predict responses to the newer FOLFIRINOX and gemcitabine/nab-paclitaxel chemotherapy regimens with the basal subtype showing poorer response particularly to FOLFIRINOX [56]. More recent work by Lomberk and colleagues has now linked the epigenetic profile of human PDA with patient outcomes [9]. Here the authors developed 24 patient-derived xenograft (PDX) models and then comprehensively characterized their genome-wide histone states, DNA methylation status and the classical versus basal-like subtype of each PDX. They found that the epigenetic classification paralleled the transcriptomic definitions of PDA types and was also prognostic for overall patient survival [9]. These studies further support the clinical translational relevance of the PDA subtype framework that now exists. Future efforts in PDA prognostic and predictive biomarker development should strive to integrate the new findings into this framework.

### 4.2. ARID1A

Components of the SWI/SNF—SWItch/Sucrose Non-Fermentable—nucleosome remodeling complexes are mutated in many PDAs [6,8,10,57]. ARID1A—the AT-rich interacting domain 1A-is frequent mutational target within SWI/SNF complexes in PDA and has been implicated in PDA initiation and development in the genetically engineered mouse models of the disease [15,16,17]. ARID1A loss of expression in human PDA portends poorer overall survival in patients [57]. Independent of the expression level, presence of an ARID1A mutation correlates with improved patient survival [58]. Functional explanation for these distinct correlations is still lacking and it is interesting to note that the mutational and expression level status for this nucleosome regulator each potentially affect PDA biology differently. As demonstrated below, this concept is applicable to other epigenetic regulators and their activity in PDA.

### 4.3. Trithorax and Polycomb Proteins

Trithorax proteins form complexes that generally increase access to chromatin and activate transcription through regulating histone post-translational modifications like histone 3 lysine 4 and 27 methylation (H3K4 and H3K27) [59]. Trithorax complex activity is balanced by the Polycomb Repressor Complex 1 and 2 (PRC1/2), which regulate chromatin compaction and associated transcriptional silencing through H3K27 methylation and histone 2A lysine 119 ubiquitination (H2AK119ub) [59]. These complexes are dysregulated and contribute to the oncogenesis of many solid organ tumors.

Members of the mixed-lineage leukemia protein family (MLL1/2/3/4) are Trithorax components and functional histone methyltransferases (KMT2A/B/C/D) that regulate H3K4 methylation and transcriptional activation. They are frequent mutational targets in PDA [6,8,10,57]. Similar to the case of ARID1A, both the mutational status and expression levels are prognostic for patient survival. MLL1, MLL2 and MLL3 mutations found in the circulating tumor DNA of PDA patients correlate with significantly better survival in one cohort of patients [58]. This was a somewhat surprising finding since MLL protein mutations were also found to correlate with the basal-like/squamous PDA subtype, which classically has worse survival [8]. How the method of MLL mutation detection—directly from tumor versus from circulating free DNA—affects these results is currently unclear. Decreased expression levels of MLL2/3 were correlated with improved patient survival in one study [60]. Mechanistically, this survival effect was linked to inhibited tumor cell proliferation and cell cycle progression. Inhibition of MLL2/KMT2D expression also sensitized PDA cells to 5-FU therapy in vitro [60]. The complexity and importance of the genomic and environmental context for MLL2 function is demonstrated by the contradictory findings of Kotsioumpa and colleagues, who defined MLL2 as a functional tumor suppressor in their PDA human models [61]. In this work, inhibition of MLL2 expression led to increased aerobic glycolysis and metabolic adaptation of tumor cells leading to increased tumor growth. Understanding how the differences in model, microenvironment and other contexts contribute to these disparate observations will yield significant insight into how MLL proteins regulate PDA aggressiveness.

Polycomb repressor complexes counteract Trithorax complex function through their regulation of H3K27 methylation and H2AK119 ubiquitination. EZH2—enhancer of zeste 2, a component of PRC2—and BMI1—a component of PRC1—are key players in PDA initiation and progression [62,63]. Both EZH2 and BMI1 have also been implicated in pancreatic cancer stem cell maintenance, a functional subset of tumor cells thought to promote PDA chemoresistance and tumor relapse [64,65]. In patients, higher levels of EZH2 and BMI1 expression correlate with poorer tumor differentiation/higher grade and worse survival [66,67]. How PRC dysregulation contributes to PDA evolution and establishment of PDA subtypes and their biology is less clear and remains an active area of study.

### 4.4. KDM6A/UTX

Lysine demethylase KDM6A/UTX cooperates with MLL proteins and removes the methyl groups from trimethylated H3K27 thereby reversing EZH2-mediated transcriptional suppression [68]. KDM6A mutations are frequent in human PDA [6,8,10] and their presence has been correlated with the development of the squamous/basal-like PDA subtype [8,18]. These findings have been recapitulated in the genetically engineered PDA mouse models, where Kdm6a genetic deletion led to activation of the ΔNp63, Runx3 and Myc oncogenes and their related transcriptional network, which have all been implicated in the establishment of the basal-like PDA subtype and increased aggressiveness [8,13]. More importantly, loss of KDM6A in human PDA cell lines increased their sensitivity to bromodomain and extra-terminal (BET) inhibitors and G9α methyltransferase inhibitors [13]. Histone acetylation status also plays a key role in transcriptional activity regulation (see below) and KDM6A-null cells appear to rely on histone deacetylase (HDAC) activity for their survival. Loss of KDM6A sensitizes these cells to HDAC inhibitors as well [13,18]. Based on these observations, PDA tumor subtypes and their epigenetic correlates can potentially serve as rational tumor biology-based predictive biomarkers for systemic therapy selection.

### 4.5. Histone Deacetylases

Histone acetyltranferases (HATs) and deacetylases (HDACs) form a key part of the transcriptional regulation machinery in PDA [69]. Their dysregulation partly contributes to the establishment of the KRAS-driven oncogenic transcriptional programs in the tumor epithelium. Over the past decade studies determined that overexpression of HDAC1 in human tumors correlates with poor patient survival [70,71]. At least part of this association may be attributed to HDAC1 regulation of the invasion and epithelial–mesenchymal transition (EMT) transcriptional programs [71]. Additionally, other known PDA-associated mutations or expression changes may modulate the sensitivity of tumor cells to HDAC inhibitors. For example, squamous PDA subtype tumors lacking KDM6A demethylase show an increased sensitivity to HDAC inhibition [13,18]. These results highlight the functional interplay between multiple epigenetic regulatory mechanisms, which may potentially be leveraged for therapy development.

### 4.6. DNA Methylation

CpG island DNA methylation is another key transcriptional regulatory mechanism. Tumor specific alterations in epithelial genomic DNA methylation form the basis of some of the early detection strategies being developed for PDA (see Section 3 above). Analysis of the DNA methylation profiles within the TCGA cohort has also defined two predominant types of tumors [6]. However, contrary to the genome-wide histone modification profiles, which correlate well with the classical/basal transcriptomic PDA types, the DNA methylation profiles form distinct groups separate from the classical/basal-like transcriptome taxonomy. The correlation of methylation status with patient survival is also locus dependent. Genome-wide analysis of DNA methylation sites identified 17,251 loci associated with increased survival and 3256 loci associated with decreased patient survival [72]. Additionally, DNA methyltransferase 1 (DNMT1) expression itself is a prognostic marker that correlates with poorer patient survival [70]. These observations implicate the importance of DNA methylation and associated transcriptional regulation in PDA carcinogenesis. However, unlike our use of DNA methylation for diagnosis, its use in clinically relevant biomarker applications remains in its infancy.

### 4.7. Histone Variants

Nucleosomes are classically composed of two copies of histone H2A, H2B, H3 and H4 each [73]. Histone H1 and its variants stabilize nucleosome complexes with the supercoiled DNA helix wrapped around the nucleosome core. One basic question is whether histone composition and variant use contribute to PDA biology and association survival. A recent mass spectrometric analysis of 10 fresh resected tumor specimens explored this question [74]. In this analysis only histone H1.3 was found to be primarily expressed in the tumor tissue compared to the normal pancreas. The authors confirmed the mass spectrometry results with tumor immunohistochemistry. Analysis of a separate cohort of 62 PDAs revealed that 12 (19.2%) tumors did not express H1.3 by immunohistochemistry and these patients survived longer than the H1.3-expressing cohort [74]. H1.3 expression also did not correlate significantly with any of the other classical pathologic prognostic factors including tumor size, differentiation, lymph node metastasis, resection margin status, or receipt of adjuvant chemotherapy, which may mark this histone variant as a possible useful prognostic marker in the future.

### 4.8. Summary and Outlook for Epigenetic Biomarkers in PDA

Although significant amount of work has clearly been performed in PDA epigenetic regulation, the development of high-quality prognostic and predictive biomarkers based on our understanding of overall PDA biology is still in its early stages. It will be important to further integrate our understanding of epigenetic regulation in pancreatic tumors with the known biological subtypes along the lines of what Lomberk and colleagues have begun to do [9]. All of the prognostic and predictive studies described above were preliminary and hypothesis-generating only (see Table 1 for summary). The hard work of prospective validation and biomarker development will necessitate the use of independent rigorously clinically annotated cohorts similar to the COMPASS trial cohort that has already begun to yield deep clinically relevant and actionable understanding of PDA biology [19,55,75].

## 5. Epigenetic-Based Therapy of Pancreatic Cancer

The previous sections have detailed our increasing understanding of how epigenetic mechanisms contribute to PDA progression, diagnosis of the disease and its outcomes. Translation of this knowledge to effective new therapies remains in early stages with promising preclinical studies pointing the way to potential new combination treatments employing epigenetic approaches. In this section, we provide a brief overview of the current state of published clinical trials targeting epigenetic regulators of PDA.

### 5.1. Histone Deacetylase Inhibitors

The prognostic role of histone acetylation in PDA has already been described earlier in this review (see Section 4.5). The biological role of HDACs in PDA has led to the development and testing of multiple HDAC inhibitors of varying specificities individually or in combination with other systemic therapies. Majority of these trials have been designed as Phase I, single-arm dose-finding studies with occasional small expansion cohorts to test for signs of efficacy [76,77,78,79,80,81,82,83,84,85,86]. These trials have primarily defined the toxicity of HDAC inhibition in PDA patients and the findings have remained remarkably consistent across inhibitors of all HDAC classes and the various combinations of other chemotherapeutic agents paired with them. Most severe treatment-related adverse events included hematological (anemia, leukopenia, thrombocytopenia), gastrointestinal (nausea, emesis, stomatitis) and constitutional (fatigue) symptoms. Dose reductions or protocol withdrawal was common in these early studies. In vast majority of these smaller, single-arm trials, the best response to therapy was only stable disease by RECIST criteria—the primary target tumor, measured during the trial, did not shrink more than 30% and could have grown as much as 20% in size. The only randomized, placebo-controlled, Phase II trial using an HDAC inhibitor in pancreatic cancer tested the oral compound CI-994 in combination with gemcitabine and compared it to gemcitabine only [76]. The combination therapy did not improve survival or objective response rate compared to gemcitabine alone and more severe toxicity was seen with the CI-994 compound.

Several potential reasons exist as to why HDAC inhibitor therapy has not been as successful as was originally hoped. HDACs comprise a large set of enzymes with four classes and different biochemical specificities. Recent preclinical work has highlighted the need to fully understand which HDACs are the relevant protumorigenic isoforms to allow for more selective targeting [87]. Recent preclinical data also suggests that HDAC inhibition targets not only the tumor epithelium, but at the same time may promote the development of tumor-supportive cancer-associated fibroblast subpopulations in the tumor microenvironment [88]. The single agent trials have also demonstrated that current HDAC inhibitors have minimal effect even at toxic doses. Another way to extend their applicability in the future is to combine them with agents targeting other epigenetic pathways like BET inhibitors or inhibitors of downstream KRAS signaling pathways including MEK, PI3K and GSK3beta [13,89,90,91]. The complexity of the histone acetylation response and its downstream targets will require much more translational work before we can rationally design HDAC inhibitor-based therapies for pancreatic cancer.

### 5.2. DNA Methyltransferase Inhibitors

Dysregulation in DNA methylation is one of the key epigenetic changes seen in PDA and its use in the diagnosis and prognosis has been described above (see Section 3.2, Section 3.3 and Section 4.6). Genomic DNA is methylated by a set of DNA methyltransferases (DNMTs) and small molecule inhibitors against these enzymes were among the first epigenetic inhibitors adopted for clinical use. Two recent Phase I trials using DNMT inhibitors in pancreatic cancer patient cohorts have been published. Von Hoff and colleagues used CC-486 (oral formulation of 5′-azacitidine) in a three-arm trial design—CC-486 alone, with carboplatin and with nab-paclitaxel—with enrollment into each arm based on the histology of the primary tumor [92]. Arm B consisted of CC-486 and nab-paclitaxel and included 24 pancreatic cancer and 22 non-small cell lung cancer patients. The combination regimen led to stable disease in 46% of these patients. No objective responses, where a tumor shrank more than 30%, were observed. Significantly better efficacy was seen in some of the other histologic subgroups and treatment arms. A total of 20 Grade 3 or 4 adverse events were seen in Arm B primarily consisting of hematologic and gastrointestinal toxicities [92]. Gaillard and colleagues used a combination of CC-486 and romidepsin (HDAC inhibitor) in a small cohort of pretreated solid tumor patients. This cohort also achieved stable disease as the best response with similar hematological and gastrointestinal adverse event profile as the earlier CC-486 trial [78]. DNMT inhibitor trials in solid organ tumors have generally been plagued by significant toxicity and poorer efficacy than in hematologic malignancies. Ongoing research aims to expand their therapeutic window by altering their specificities and mechanism of action.

A rapidly developing area of translational research focuses on the ability of DNMT inhibitors to reactive the latent anti-tumor response [93]. The underlying mechanisms triggered by DNA demethylation increase tumor antigen, immune checkpoint ligand and receptor expression and reactivation of endogenous retroviral sequences leading to a Type I anti-viral interferon signaling response [94,95]. These findings have led to ongoing trials of demethylating therapy along with immune checkpoint inhibitors in a variety of solid organ tumors including pancreatic cancer. Whether this approach will be more effective remains to be seen.

### 5.3. Bromodomain and Extraterminal Domain (BET) Protein Inhibitors

Significant amount of preclinical evidence for efficacy of BET protein inhibition in PDA exists in a variety of systems. BETi compounds have demonstrated efficacy against basal-like/squamous subtype of PDA cells representing a potentially powerful way to treat more advanced and aggressive pancreatic tumors [13]. The well-studied compound JQ1 functions as a BET inhibitor and has been tested directly in PDA xenograft models with activity partly related to its ability to control cell cycle progression through the CDC25B phosphatase [96]. As with other epigenetic approaches, investigators have also studied combination treatments using BETi and other epigenetic modifiers. An example of this approach was the pairing of JQ1 and Vorinostat/SAHA, which exhibited synergistic effect in killing pancreatic cancer cell lines in vivo, treating autochthonous murine tumors in the *Ptf1a-Cre, Kras^+/LSL-G12D^, Trp53^loxP/loxP^* (KPC) model of PDA and controlling primary human xenograft tumors [89]. Another combination approach, which was tested in solid tumor-derived cell lines not including the pancreas, combines BETi with CDK9 inhibitors to counteract the BRD4-dependent upregulation of MYC during solo CDK9 inhibition [97]. Further work will have to determine how applicable this approach is in pancreatic cancer.

Despite these preclinical findings, there are currently no published clinical trials using BETi systemically in pancreatic cancer despite multiple trials being registered in the past [98]. However, early experience with BET inhibition in other solid organ tumors has been published. Lewin and colleagues studied the novel BRD2/3/4 inhibitor birabresib in patients with non-small cell lung cancer, castration-resistant prostate cancer and nuclear protein in testes (NUT) midline carcinoma [99]. In this trial 46 patients were enrolled with 42 evaluable for efficacy. The primary adverse events noted were hematologic and gastrointestinal with 83% of patients suffering some treatment-related adverse event and 35% having serious (Grade 3 or 4) adverse events. Seven percent of patients demonstrated a partial response (tumor shrinkage > 30%) and another 60% had stable disease—tumor shrank less than 30% and did not increase in size more than 20%. A second Phase I trial of the Bayer compound BAY1238097 had to be stopped early due to significant dose-limiting toxicities [100]. Of the eight enrolled patients four were evaluable for efficacy. Two demonstrated stable disease and the other two progressed on treatment. The authors hypothesized that the poor tolerance of the compound was partly due to off-target toxicity not necessarily related to the BET inhibition. Whether similar results will be seen in pancreatic cancer patients remains unclear.

### 5.4. EZH2 Inhibitors

As we noted earlier, a subset of PDA tumors carries mutations in the SWI/SNF chromatin remodeling complex components (see Section 4.2 above). In oncogenic contexts, loss of SWI/SNF function tends to promote chromatin reprogramming by the EZH2-mediated histone 3 lysine 27 trimethylation, which in turn supports tumorigenesis [101,102]. Interestingly, more recent work has defined an enzymatic-independent component to this process, which relies on the maintenance of the structural integrity of the EZH2-containing Polycomb Repressor Complex 2 (PRC2) [102]. These findings suggested that SWI/SNF-deficient tumors are oncologically addicted to EZH2 function and, among others, have led to efforts to target EZH2 with small molecule inhibitors.

Although no Phase II or III trials have been reported yet, two Phase I trials of EZH2 inhibitors are now published. In 2018, Italiano and colleagues reported the results of the Phase I trial of tazemetostat in 64 patients, including 43 patients with relapsed or refractory solid tumors [103]. They identified 13 patients in the solid tumor group with mutations in their SWI/SNF components. All four patients with a partial response to the treatment in this trial fell into this subgroup. Some of the responses were prolonged for over a year. Additional five patients demonstrated stable disease with minimal tumor enlargement. Nine percent of the entire treated cohort suffered Grade 3 or 4 treatment-related adverse events. Overall, these results support the concept, demonstrated by preclinical studies, that SWI/SNF-deficient tumors rely on PRC2 function to support tumorigenesis. This suggests that SWI/SNF component mutation and expression status may serve as a biomarker for future EZH2 inhibitor trials.

A Phase I trial using the novel EZH2 inhibitor GSK2819126 was reported in 2019 [104]. Forty-two patients, including twenty-one with solid tumors with two being pancreatic cancers, were enrolled. In this trial 67% of patients demonstrated stable disease but no partial or complete responses were observed. It is unclear whether the two patients with PDA progressed or remained stable. No assessment of SWI/SNF status was performed in this cohort to allow for biomarker stratification of the patients. Thirteen patients suffered Grade 3 or 4 adverse events, but none were deemed related to the treatment itself. These two trials established the relative safety of EZH2 inhibitors in the treatment of solid organ malignancy and, together with stratification based on SWI/SNF mutation status, could form a viable epigenetic treatment approach for patients with PDA.

Overall, epigenetic approaches to PDA treatment are slowly progressing forward with multiple trials opening and beginning to recruit patients across the world (Table 2). These now include single agent trials as well as combinations with standard-of-care chemotherapy and immunotherapy. As our understanding of PDA biology increases, these will likely become a standard part of our treatment armamentarium in the future.

## 6. Conclusions

Epigenetic regulation plays a key role in pancreatic cancer initiation and progression. Our understanding of these processes is beginning to provide us with a clearer picture of how to best diagnose, stratify risk and treat our patients. The development of new diagnostic tests will most likely depend on the isolation and identification of multiple types of analytes from easily accessible sites or body fluids including blood, urine and saliva. Epigenetic markers such as aberrant methylation of circulating tumor DNA should play a key role in these multipronged assays. Circulating tumor cells are another plausible target in pancreatic cancer and their epigenetic profiling may yield additional insights into pancreatic tumor biology, its diagnosis and treatment. Specific epigenetic and chromatin regulator mutations and expression changes will also likely serve as predictive biomarkers for more targeted systemic therapy selection. It will be important to combine these insights with our understanding of intratumoral heterogeneity, tumor evolution and treatment resistance mechanisms to define the most effective chemotherapy and radiation combinations. Significant amount of work remains, but the future for epigenetic research and its potential to help our patients is bright.

## Figures and Tables

**Table 1:** Epigenetic regulators, their association with PDA subtypes and their survival and therapeutic relevance.

	Epigenetic Biomarker	Function	PDA Sub-Type Association	Survival Impact	Chemoresistance
1	DNA methylation status	Hypomethylation—active gene transcription Hypermethylation—gene repression	Classical Basal	Better survival Worse survival	
2	CpG island methylation			Locus dependent	Variable depending on loci involved
3	ARID1A	Component of SWI/SNF complex (transcriptional activator)		Poor when ARID1A is mutated	Loss of ARID1A may increase sensitivity to PARP inhibitors
4	MLL (1/2/3/4)		Basal-like/ squamous	Improved in cases of mutation or low expression	5-FU sensitization with MLL2 inhibition
5	BMI1	Component of PRC1—repression of gene expression (chromatin compaction)		Decreased survival with overexpression	Increased
6	EZH2	Component of PRC2–H2K27 methylation—gene repression		Decreased survival with overexpression	Increased
7	KDM6A/UTX	Lysine demethylase (gene expression)	Basal-like/ Squamous		KDM6A loss associated with HDAC inhibitor sensitization
8	HDACs	Transcriptional machinery components		Poor survival with high HDAC1 expression	

**Table 2:** Active clinical trials in pancreatic cancer focusing on epigenetic diagnosis, monitoring and therapy (clinicaltrials.gov).

	Title	Status	Study Results	Conditions	Interventions
1	Determination Safety and Tolerability of Epigenetic and Immunomodulating Drugs in Combination With Chemotherapeutics in Patients Suffering From Advanced Pancreatic Cancer. **NCT04257448**	Recruiting	No results available	Pancreatic cancer	Romidepsin (HDACi) Azacitidine (methylation inhibitor) nab-Paclitaxel Gemcitabine Durvalumab Lenalidomide
2	p53/p16-Independent Epigenetic Therapy With Oral Decitabine/Tetrahydrouridine for Pancreatic Cancer **NCT02847000**	Completed	No results available	Metastatic pancreatic cancer	Tetrahydrouridine Decitabine (methylation inhibitor)
3	Trial to Improve Outcomes in Patients With Resected Pancreatic Cancer (Azacitidine, Abraxane, Gemcitabine) **NCT01845805**	Active, not recruiting	No results available	Pancreatic cancer	Azacitidine (methylation inhibitor) First-line chemotherapy after recurrence
4	Combining Epigenetic And Immune Therapy to Beat Cancer. **NCT04705818**	Recruiting	No results available	Advanced Solid Tumor Advanced Colorectal Carcinoma Advanced Soft-tissue SarcomaAdvanced Pancreatic Adenocarcinoma Adult Solid Tumor	Durvalumab Tazemetostat (EZH2i)
5	Circulating Epigenetics in Pancreatic Surgery **NCT04947696**	Recruiting	No results available	Pancreatic neoplasms	Blood sampling for diagnosis
6	Superenhancer Inhibitor Minnelide in Advanced Refractory Adenosquamous Carcinoma of the Pancreas (ASCP) **NCT04896073**	Recruiting	No results available	Adenosquamous carcinoma of the pancreas	Minnelide
7	Blood Sample Collection to Evaluate Biomarkers in Subjects With Untreated Solid Tumors **NCT03662204**	Recruiting	No results available	Breast Cancer Lung Cancer Colorectal Cancer Prostate Cancer Bladder Cancer Uterine Cancer Kidney Cancer Renal Pelvis Cancer **Pancreatic Cancer** Liver Cancer Stomach Cancer Ovarian Cancer Esophageal Cancer	Blood sampling

## Data Availability

This literature review did not report any new data that would require specific access.
